# Pharmaceutical industry payments to NHS trusts in England: A four-year analysis of the Disclosure UK database

**DOI:** 10.1371/journal.pone.0290022

**Published:** 2023-11-01

**Authors:** Piotr Ozieranski, Eszter Saghy, Shai Mulinari

**Affiliations:** 1 Department of Social and Policy Sciences, University of Bath, Bath, United Kingdom; 2 Division of Pharmacoeconomics, Faculty of Pharmacy, University of Pecs, Pecs, Hungary; 3 Department of Sociology, Lund University, Lund, Sweden; UCL: University College London, UNITED KINGDOM

## Abstract

**Introduction:**

Although hospitals are key health service providers, their financial ties to drug companies are little understood. We examine non-research pharmaceutical industry payments to English National Health Service (NHS) trusts—hospital groupings providing secondary and tertiary care.

**Methods:**

We extracted data from the industry-run Disclosure UK database, analysing it descriptively and using the Jonckheere-Terpstra test to establish whether a statistically significant time trend existed in the median values of individual payments. We explained payment value and number per trust with random effects models, using selected trust characteristics as predictors.

**Results:**

Drug companies reported paying £60,253,421 to 234 trusts, representing between 90.0% and 92.0% of all trusts in England between 2015 and 2018. As a share of payments to all healthcare organisations, the number of payments rose from 38.6% to 39.5%, but their value dropped from 33.0% to 23.6%. The number of payments for fees for service and consultancy and contributions to costs of events increased by 61.5% and 29.4%. The median payment value decreased significantly for trusts overall (from £2,250.8 to £1,758.5), including those with lower autonomy from central government; providing acute services; and from half of England’s regions. The random effects model showed that acute trusts received significantly more money on average than trusts with all other service profiles; and trusts from East England received significantly less than those from London. However, trusts enjoying greater autonomy from government did not receive significantly more money than others. Trusts also received significantly less money in 2018 than in 2015.

**Conclusion:**

NHS trusts had extensive pharmaceutical industry ties but were losing importance as payment targets relative to other healthcare organisations. Industry payment strategies shifted towards events sponsorship, consultancies, and smaller payments. Trusts with specific service and geographical profiles were prioritised. Understanding corporate payments across the health system requires more granular disclosure data.

## Introduction

Since 2013 the government-run US Open Payments database has informed extensive research on individual-level conflicts of interest (COIs) associated with pharmaceutical industry payments for collaborations with physicians [1], with key areas of interest including association between payments and prescription of marketed drugs [2–5]; and payments underreporting, for example, by clinical triallists [6], authors of clinical practice guidelines [7–9], and researchers [10, 11]. However, because Open Payments covers only payments to physicians (and since 2021) nurses, it has informed few studies on institutional COIs, understood as situations when a secondary interest associated with, for example, accepting external funding, risks compromising the organisation’s primary aims, obligations, mission or specific activities [12, 13]. While institutional COIs can affect multiple healthcare organisations (HCOs), including academic [14–16] and public health education intuitions [17], Open Payments covers only one HCO type, teaching hospitals [18], that is “any hospital anywhere in the United States or its territories, receiving Medicare, as well as indirect or direct graduate medical education payments” [19].

Despite being a major recipient of pharmaceutical and medical device industry payments, worth almost $2bn in 2021 alone [20], payments to teaching hospitals, and the associated potential institutional COIs, have only attracted a handful of studies. For example, in 2018, 90% of the studied teaching hospitals accepted payments from biomedical companies, primarily royalties, including research collaborations with drug companies, and education grants [21]. These payments were significantly associated with hospital size, connections with major medical schools, and service quality [21]. Another study found evidence of increasing payments to cancer centres from 2013 to 2019, which were correlated with payments to oncologists working at organisations receiving payments [22]. A third study found that in 2018, 31 companies paid $7m—mostly for service fees—to teaching hospitals in relation to the marketing of opioid products [23].

In contrast to the US government regulation, the possibilities for studying institutional COIs should be much greater in Europe, where payment disclosure is governed predominantly by pharmaceutical industry self-regulation [24, 25]. This is because these disclosure requirements cover, in principle, the entire spectrum of HCOs forming the health system, including the public, private, and non-profit sectors, and ranging from healthcare providers, commissioners, regulators to professional bodies [26]. Importantly, payments to HCOs are typically unaffected by the European pharmaceutical industry’s precautionary interpretation of privacy rights, allowing individual recipients to refuse public payment disclosure [27, 28]. Nevertheless, in most countries efficient analysis of payments to HCOs is precluded by the “closed” electronic formats of disclosures, published as portable document files (PDFs), and their dispersal on multiple drug company websites [24, 25]. Further, even centralised databases, such as the industry-run Disclosure UK [29] or Irish transferofvalue.ie [30], suffer from the absence of built-in recipient categories and the lack of recipient disambiguation [24, 26, 31, 32].

Unsurprisingly, then, no research has examined, as far as we are aware, drug company payments to HCOs in Europe, except for a UK-wide overview [26, 33], followed by case studies of several HCO types in England, the largest of the four constituent UK countries. This body of work has revealed extensive pharmaceutical industry payments among the major pillars of the healthcare system [34, 35]: general practices [36], constituting the first point of contact for most patients (primary care); clinical commissioning groups (CCGs) [37], tasked with the regional planning and procuring of health services; and NHS trusts [38], the key organisational units involved in providing accident and emergency services as well as secondary and/or tertiary care free at the point of delivery, typically following patient referrals by general practices.

Given the practical challenges associated with extracting and processing data from Disclosure UK [26, 32], we choose to focus on NHS trusts, identified, among other secondary and tertiary care providers, as the recipients of the largest share of payments in the UK in 2015 [26]. Further, an investigation of “joint working” payments to NHS trusts, involving pooling resources by drug companies and service providers, has revealed important deficiencies in the transparency of these collaborations [38].

There are currently around 217 NHS trusts in England [39], with many managing several hospitals [40]. They currently employ 0.8m staff and generate £104bn of annual spending [41, 42] which is covered by general taxation, supplemented by national insurance contributions [43]. As of 2020/21, medicines dispensed in NHS trusts cost £7.59bn, representing 44.3% of the total drug expenditure in England [44]. They are also largely responsible the growing medicines spending, with 12% average yearly increases between 2010/11 and 2016/17 [45].

Based on the type of health services provided, NHS trusts are traditionally divided into acute (specialised health services and emergency services), ambulance (stabilising patients and transporting them to hospitals), mental health (psychological and psychiatric care), and community (supporting people to live independently by providing a range of care including home visits) [46]. A new type of NHS trusts are “integrated care” organisations, joining up primary and secondary care with social, dental, pharmacy and optician services within a single provider [47, 48]. While acute trusts are by far the dominant type [49], the NHS long-term strategy promotes the expansion of community health services [50] and integrated care organisations [51]. As for their governance, NHS trusts fall into non-foundation trusts and foundation trusts, with the latter enjoying greater autonomy from central government in planning and delivering local health services [52, 53]. All NHS trusts are encouraged to apply for the foundation trust status, with around 145 out of the 217 (66.82% of the total number) having it authorised [54]. Nevertheless, establishing foundation trusts has been hindered in recent years by declining funding and a broader trend towards increased government control of healthcare provision [55].

In studying pharmaceutical industry payments to NHS trusts, we seek to contribute to the scholarship on collaborations between drug companies and healthcare actors in two ways. First, while increasingly popular in studies of US physicians [56–59], longitudinal studies of drug company payments are practically impossible in a vast majority European countries, given the challenges in data availability and accessibility characterised above. Therefore, Disclosure UK, a centralised payments database, offers a unique opportunity to begin establishing a longitudinal agenda in European research on pharmaceutical industry payments to HCOs. Second, an emerging body of work in the US examines recipient, primarily physician, characteristics [60–62] that might explain the patterns of industry payments, therefore providing novel insights into drug company marketing strategies. This line of research has been undertaken at the organisational level in England, identifying that larger general practices, those with higher shares of elderly patients, and those located in less deprived areas, received significantly higher median payment values [36]. In addition, practices based in northern regions of England had significantly higher median payments than those based in London [36].

Against this background, we sought to examine the (1) distribution and (2) trends in pharmaceutical industry payments to NHS trusts in England; as well as (3) ascertain whether NHS trust organisational and geographic characteristics were associated with industry payments.

## Materials and methods

We conducted a retrospective review of payments to NHS trusts in England reported in Disclosure UK, combining the payment data with other data sources. We selected the NHS trust as our unit of analysis for two reasons. First, they manage their constituent hospitals and ordinarily strategic decisions of interest to drug companies would be taken at the trust level. Second, payment recipients identified as hospitals in Disclosure UK could be linked with NHS trusts but not the other way round; taking this approach therefore helped minimise the loss of data.

### Data extraction and cleaning

The data extraction took place between June 2019 and July 2020 using a structured protocol (S1 Appendix in S1 File). It was developed by PO together with ES based on our previous research [26, 36], and executed by ES. The protocol underwent several alterations and refinements to maximise the accuracy and consistency of data extraction in light of emerging data quality issues. Overall, the changes involved adding and triangulating approaches to identifying NHS trusts; distinguishing NHS trusts from other organisational payment recipients; and standardising NHS trust names across companies and years. We introduced changes to the protocol iteratively and applied them systematically to all relevant data. We present the final version protocol integrating all the rounds of changes made throughout the data extraction.

We considered four yearly versions of Disclosure UK from 2015 to 2018 downloaded from the website of the industry trade group, the Association of the British Pharmaceutical Industry (ABPI) [63]. The inclusion of the two more years that became available over the course of data extraction and analysis was not feasible, given the amount of work involved in data processing.

Each Disclosure UK dataset comprised payments reported by ABPI members and other companies subscribing to the ABPI Code of Practice for the Pharmaceutical Industry (henceforth: ABPI Code) voluntarily, thereby constituting the most comprehensive source of insight into financial relationships between the pharmaceutical industry and the UK healthcare sector [64]. As payments associated with drug company research and development (R&D) activities do not include named recipients, we only considered non-R&D payments, i.e., grants and donations, fees for service and consultancy, event sponsorship, and joint working (projects involving pooling together resources by HCOs and drug companies) [26].

**Fig 1:**
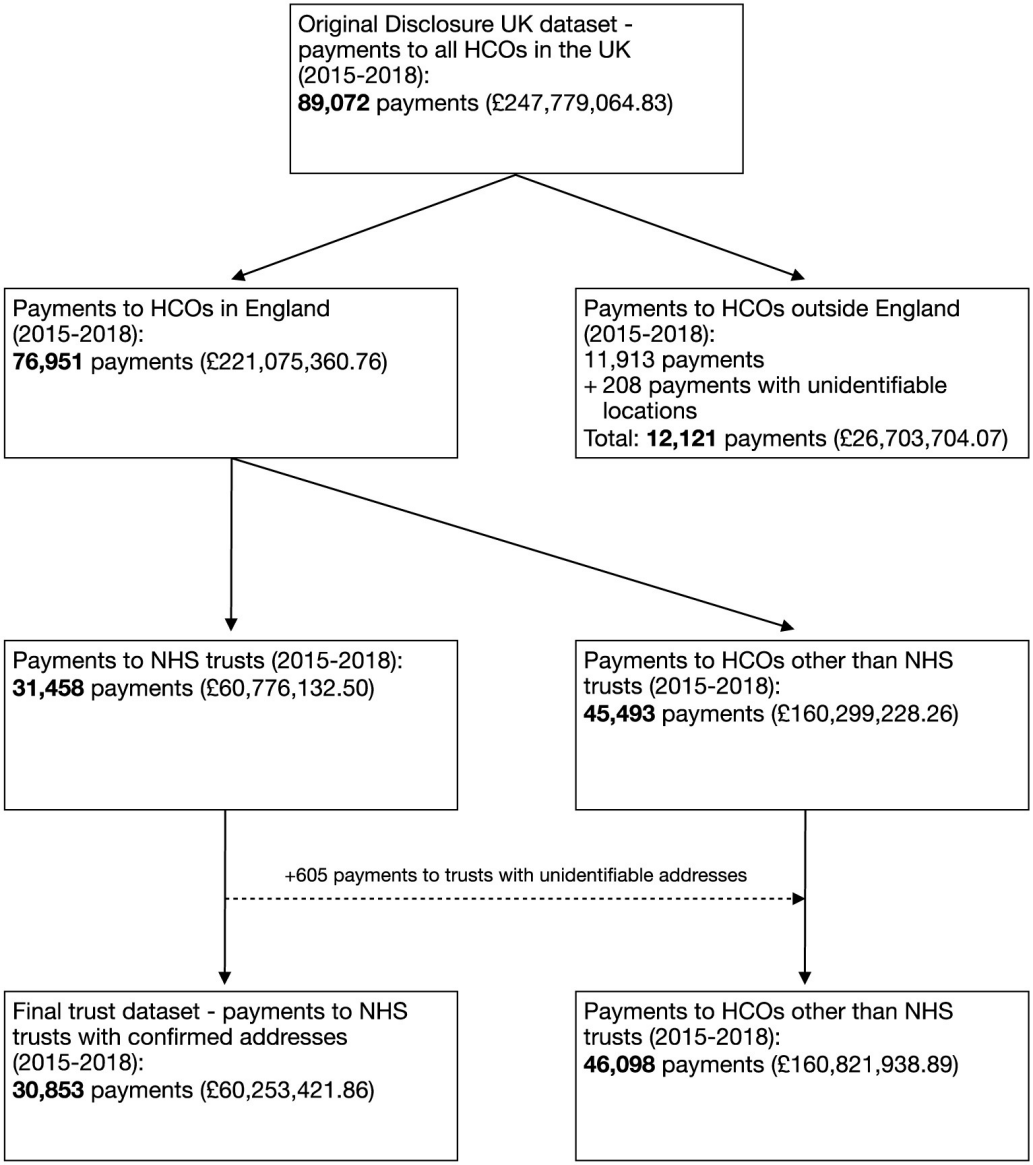
Summary of data extraction. **Notes**: Abbreviations: NHS = National Health Service; HCOs = healthcare organisations. Values in brackets are value of payments adjusted by inflation and VAT approaches taken by companies.

Disclosure UK includes payments reported to HCOs in the entire UK and therefore we excluded payments to HCOs made outside of England. We did so by linking the recipients’ official postcodes reported in Disclosure UK to their geographical location, including all UK nations, islands and the eight regions of England. The prefixes, i.e. the letter(s) at the beginning of the postcode, were extracted for each postcode and matched to a dataset with the region each prefix stands for [65]. We excluded payments made to HCOs in the Channel Islands (n = 48), Ireland (n = 2), Isle of Man (n = 7), Northern Ireland (n = 1,393), Scotland (n = 5,751), Wales (n = 4,712), as well as those with unidentifiable postcodes (n = 208). These checks reduced the number of payments to HCOs in our database from 89,072 to 76,951 (Fig 1).

As Disclosure UK does not categorise HCOs by type, we identified NHS trusts through detailed online searches involving information reported in the following Disclosure UK columns: (1) institution name, (2) location (unstandardised reporting, typically an organisational sub-unit), (3) street address, and (4) postcode [26, 36]. Alongside this, we standardised the NHS trust names to account for differences in naming by the same and different companies [26, 36]. To this end, we created an Excel spreadsheet with a full list of official NHS trust names obtained from the official NHS website [66]. We used the Wayback Machine digital internet archive [67] to extract trust lists from previous years and thus included trusts which had gone through a name and/or organisational merges. From each official trust name, we derived one, or, in some cases, two search terms that were subsequently applied to Disclosure UK. In instances when an NHS trust could be identified using the search terms were recorded it in an additional spreadsheet column including its official (or standardised) name. We inserted the standardised NHS trust name to this column only if the trust name could be evidently identified based on searches in the institution name, location, or address column.

Drawing on our previous research [26], we anticipated that some NHS trusts would be identified at a lower level of aggregation, i.e., by the names of their constituent hospitals. Therefore, we used “hosp” as an additional search term for the second round of searches. We connected hospitals to trusts using the official NHS website, which includes a complete list of hospitals and their NHS trusts [66]. The name checks associated with trust and hospital names resulted in 31,458 payments identified as made to NHS trusts in Disclosure UK.

Because we used data reported by different companies over time, and because we found several instances when a reported trust name and address did not match its official address, we conducted a data quality check to ascertain whether the postcodes provided in Disclosure UK indeed belonged to the NHS trusts listed in the dataset. In so doing, we collected the main official postcode for each NHS trust from the official NHS website [66] because presumably those were used the most by companies reporting their payments in Disclosure UK. Any postcode found in the database that did not match the official postcode of the trust was checked individually to establish whether it could be linked clearly to an NHS trust. If further details of the address could not be confirmed to be part of the trust based on the NHS website, the payment was excluded from the analysis. These checks led to the exclusion of 605 additional payments.

We took the total number of NHS trusts for each year from a summary of NHS accounting data published by the UK government and NHS England [66]. Because some of the NHS trusts in our dataset changed names and/or merged with other NHS trusts during the period of observation, we used the names of the trusts that existed at the time when the payments were made. We established the list of merged NHS trusts based on the official NHS Digital website on organisational changes (S2 Table in S1 File). As most trusts not only changed names but merged with others, accounting for these organisational changes in the analysis was not possible; therefore, we aggregated payments at the trust level, solely considering the name of the trust, as it was recorded in the year the payment was made.

We also collected several NHS trust characteristics using annual accounts published by each trust on its website between 2015 and 2018 or obtained using freedom of information requests in instances when no publicly available report could be found. We divided trusts into foundation trusts and non-foundation trusts for the entire period of observation (there were no examples of any new foundation trusts being established). We also put NHS trusts into five categories according to the type of services provided: (1) acute trusts (secondary and tertiary health services); (2): mental health trusts (health services for patients with mental health disorders); (3) ambulance trusts (ambulance services provided to other NHS trusts); (4) community health trust (community health services); (5) integrated care trust (multiple types of services). In each case, the service profile was determined through qualitative searches involving key words, such as “community”, “acute”, “mental”, “ambulance” and “integrated”, as well as and looking for evidence of the type of service that the trust provided.

We present the dataset with extracted payments to NHS trusts in England as Online Supplement 1 in S1 Data. Payments to other HCOs in England can be accessed in Online Supplement 2 in S1 Data. The datasets include numbered payments whose order can be matched with the original Disclosure UK datasets from 2015–18.

### VAT and inflation adjustments

We adjusted the data consistently with the approach developed in our previous research [26, 36] to ensure that it was comparable across companies and time.

The ABPI Code allows companies to choose their approach to Value Added Tax (VAT) reporting freely, only requiring them to report this information in “methodological notes” published separately from the Disclosure UK database [26]. To standardise the payment values, we downloaded the methodological notes of all companies reporting payments to HCOs in the UK [63]. We extracted information on their approaches to VAT reporting by searching for the words “VAT”, “tax”, “gross” or “net”. For each company and each year, we denoted whether (1) the payments were net, i.e. exclusive of VAT, or (2) if the payments were gross, i.e. inclusive of VAT. Payments reported without VAT were not modified, while those reported with VAT were multiplied by 0.8, leading to net payments throughout the dataset.

We obtained the consumer price index (CPI) from the Office of National Statistics for each year starting from 2015 with the base value of 100 [68]. All payments reported in this article are expressed in the 2018 sterling.

### Data analysis

We analysed the payment distribution descriptively using medians with interquartile ranges, means, and standard deviations, calculating them at the level of individual payments. We conducted separate calculations for NHS trusts with a different health service focus (acute, mental health, ambulance, community, integrated care), the degree of organisational autonomy (foundation and non-foundation trusts) and located in the eight regions of England.

We examined changes in the median value of individual payments in subsequent years between 2015 and 2018 (ordinal independent variable). We compared the trends in payments to (1) NHS trusts with other HCOs; (2) NHS trusts with different organisational characteristics; and (3) across the different payment categories reported by drug companies. We evaluated the statistical significance of trends in median values using the Jonckheere-Terpstra test for ordered alternatives, considering only the trusts identified as receiving industry payments at least in one year during the period of observation [69, 70]. All assumptions required to carry out the test were met [71]. Given the lack of prior research allowing for formulating hypotheses, we tested whether payments were significantly increasing or decreasing. We also looked for significant differences between individual years, in this case considered as a categorical variable, using the Mann-Whitney U-tests adjusted with Bonferroni correction for multiple comparisons [72]. The first year (2015) was the reference for pairwise comparisons. We set the significance level at .05.

We also created random-effects regression models to establish which of the NHS trust characteristics were associated with higher payments received by NHS trusts, taking into account annual variations. We built the models separately for two outcome variables: the sum of the number and the value of payments to each NHS trust. Both outcome variables were log transformed due to their extreme skewness. The explanatory variables were the service focus, the degree of organisational autonomy, region, and year. We conducted a Breusch–Pagan Lagrangian multiplier (BPLM) test [73] to evaluate the need of a random-effects model (REM) over ordinary least squares (OLS). The null hypothesis, i.e. the variance of the random effect is zero, was rejected both in the model measuring effects on the value of payments (chisq = 857.18, p = <0.001) and the model examining the number of payments (chisq = 976.12, p = <0.001), which meant that a pooled OLS was an insufficient estimator for our models. Given that none of our explanatory variables relating to organisational characteristics varied over time, the choice of random-effects over a fixed-effects model was necessitated [74], making a Hausman test unnecessary. The original data was unbalanced as not all NHS trusts from our dataset received payments every year. Therefore, to run the models on a balanced panel data, we added rows with £0 payments for the specific years within our study period when certain NHS trust, although having received payments in other years, did not received any payments. We then conducted regression analyses on payments to the NHS trusts which received at least one payment between 2015 and 2018, setting the significance level at .05.

The analysis was conducted in RStudio 2022.07.1+554 using packages *dplyr*, *DescTools*, *stringr*, *sqldf*, *tidyverse*, *plm*, *car*, *gplots*, *tseries*, *lmtest*. We provide the details of the R codes used to conduct all calculations in Online Supplement 3 in S1 Data.

## Findings

### Donors and recipients

Between 2015 and 2018, 116 companies, representing 71.17% of all companies committing to report their payments in Disclosure UK, made non-R&D payments to NHS trusts in England. Over this period, the number of companies making payments increased from 83 of 109 (76.15%) to 96 of 122 (78.69%) (S3 Table in S1 File). Of the 116 companies making payments to NHS trusts, 65 (56.03%) did so consistently throughout the period of observation.

Overall, 234 NHS trusts in England received at least one payment, with 195 (83.33%) receiving them consistently from 2015 to 2018. The total number included 107 (45.73%) acute trusts, 72 (30.77%) integrated care trusts, 29 (12.39%) community trusts and 19 (8.12%) mental health trusts and 7 (2.99%) ambulance trusts. Separately, there were 158 (67.52%) foundation trusts and 76 (32.48%) non-foundation trusts. The number of recipients in individual years was 214, 221, 214, 210 in 2015, 2016, 2017 and 2018, respectively. These NHS trusts represented 89.54% (2015) [75], 90.53% (2016) [76], 91.45% (2017) [77], 91.70% (2018) [78] of the total number of NHS trusts in the respective years.

### NHS trusts compared to other healthcare organisations

NHS trusts received a total of 30,853 payments worth £60,253,421.86, which represented, respectively, 40.09% and 27.55% of the total number and value of payments to HCOs in England, including, among others general practices, bodies commissioning healthcare services, research institutes, universities as well as professional organisations [26] (Table 1, see also S4 Table in S1 File). Payments to NHS trust had a lower median value than payments to non-trust HCOs (£212.00 vs £314.85).

**Table 1:** Drug company payments to NHS trusts and other healthcare organisations, 2015–2018.

Healthcare organisation category	Year	Value of payments	Number of payments	Mean	Standard deviation	Median yearly payment (IQR)	P-value
**NHS trusts**	2015	£14,668,424.16	6,517	£2,250.79	£10,344.22	£254.40 (£152.64-£530.00)	*Ref*
	2016	£16,245,811.25	8,200	£1,981.20	£9,381.53	£209.90 (£125.94-£419.80)	**<0.001**
	2017	£15,452,017.40	8,239	£1,875.47	£10,859.11	£204.63 (£122.78-£491.12)	**<0.001**
	2018	£13,887,169.05	7,897	£1,758.54	£8,536.81	£233.98 (£150.00-£500.00)	**<0.001**
	All years	£60,253,421.86	30,853	£1,952.92	£10,565.24	£212.00 (£135.06-£491.12)	*NA*
**Other healthcare organisations**	2015	£ 29,761,953.05	10,349	£ 2,875.83	£ 19,149.48	£312.70 (£192.92-£866.02)	*Ref*
	2016	£ 37,857,662.39	11,490	£ 3,294.84	£ 17,227.27	£293.86 (£198.51-£785.03)	**<0.001**
	2017	£ 48,277,312.63	12,153	£ 3,972.46	£ 27,492.26	£327.41 (£204.63-£942.95)	**0.010**
	2018	£ 44,925,010.82	12,106	£ 3,710.97	£ 17,917.30	£336.00 (£200.00-£1,197.60)	0.396
	All years	£160,821,938.89	46,098	£3,488.70	£20,976.31	£314.85 (£200.00-£921.40)	*NA*

Notes

• NHS trusts in England: A Jonckheere-Terpstra test showed a significant decrease in median payments with increasing years (p <0.001).

• Other HCOs (excluding NHS trusts): A Jonckheere-Terpstra showed a significant increase in median payments with increasing years (p <0.001).

• Pairwise Mann-Whitney U-tests compared 2016, 2017 and 2018 to 2015, with the Bonferroni correction applied to P values.

• P values below 0.05, indicating statistical significance, are highlighted in bold.

Between 2015 and 2018 the number of payments to NHS trusts increased by 21.18% (Table 1). The value of payments increased by 10.75% between 2015 and 2016, but it then fell, with the overall decrease of 5.33%. Both the number and value of payments to other HCOs increased, by 50.95% and 16.98%, respectively. Consequently, the number of payments to NHS trusts as a share of payments to all HCOs grew from 38.64% to 39.48%; however, the share of payment value dropped from 33.01% to 23.61% (S4 Table in S1 File).

The decreasing value of payments to NHS trusts combined with the increasing number of payments helps explain the trend towards significantly decreasing median payment values (p <0.001), with the median dropping by 8.03% from 2015 to 2018 (Table 1). We also detected significant differences in the median value of payments made in 2015 and in the following years. By contrast, the increasing values of payments to other HCOs were reflected by significantly increasing median payment values (p <0.001), including significant differences between 2015 and 2016 and 2017.

### Payment categories

Payments to NHS trusts were made predominantly as contributions to costs of events (75.02%), followed by grants and donations (18.05%) (Table 2). Conversely, grants and donations accumulated the largest payments value (50.79%) and were followed by contributions to costs of events (26.86%). Contributions to costs of events were also associated with the lowest median payment value for the entire period (£209.90), while joint working, the smallest payment category in terms of the number of payments, had the median value almost 50 times higher (£10,221.95).

**Table 2:** Categories of drug company payments to NHS trusts, 2015–2018.

Payment category	Year	Number of payments	Value of payments	Mean	Standard deviation	Median yearly payment (IQR)	P-Value
**Grants and donations**	2015	1,129	£8,715,711.63	£7,719.85	£17,689.51	£1,562.44 (£254.40-£7,479.36)	*Ref*
	2016	1,707	£8,815,628.43	£5,164.40	£14,202.79	£136.02 (£30.23-£2,868.77)	**<0.001**
	2017	1,534	£7,766,289.66	£5,062.77	£15,914.46	£184.17 (£14.73-£2,573.26)	**<0.001**
	2018	1,223	£6,323,961.45	£5,170.86	£14,241.57	£612.00 (£70.64-£3,002.88)	**<0.001**
	All years	5,593	£32,333,599.38	£5,781.08	£16,536.49	£480.67 (£38.62-£3,964.40)	*NA*
**Contributions to costs of events**	2015	5,099	£3,171,315.70	£621.95	£3,604.75	£212.00 (£141.30-£373.50)	*Ref*
	2016	6,039	£3,682,292.18	£609.75	£2,579.20	£209.90 (£141.05-£341.09)	**0.007**
	2017	6,108	£4,122,172.64	£674.88	£3,946.69	£204.63 (£145.80-£409.27)	1.000
	2018	5,996	£4,104,790.46	£684.59	£3,635.67	£200.00 (£150.00-£400.00)	1.000
	All years	23,242	£17,100,653.47	£735.77	£5,119.74	£209.90 (£146.93-£377.82)	*NA*
**Fees for service and consultancy**	2015	247	£946,748.80	£3,832.99	£14,427.52	£795.00 (£318.00-£2,827.12)	*Ref*
	2016	406	£1,940,313.02	£4,779.10	£19,540.33	£377.82 (£128.08-£1,574.05)	**<0.001**
	2017	551	£1,300,295.52	£2,359.88	£10,378.64	£245.56 (£122.78-£982.24)	**<0.001**
	2018	613	£1,529,435.48	£2,495.00	£12,496.48	£250.00 (£110.00-£1,000.00)	**<0.001**
	All years	1,817	£6,172,556.66	£3,397.11	£14,818.02	£327.50 (£132.66-£1,266.91)	*NA*
**Joint working**	2015	80	£1,834,613.05	£22,932.66	£40,138.77	£10,759.50 (£4,538.09-£23,124.64)	*Ref*
	2016	58	£1,807,520.56	£31,164.15	£41,984.48	£11,481.58 (£4,729.59-£37,519.80)	1.000
	2017	93	£2,263,215.87	£24,335.65	£53,553.83	£8,441.12 (£2,111.09-£20,463.32)	1.000
	2018	97	£1,928,981.66	£19,886.41	£29,778.38	£9,930.42 (£4,827.50-£21,332.80)	1.000
	All years	328	£8,048,767.76	£24,538.93	£42,593.78	£10,221.95 (£4,092.66-£24,751.40)	*NA*

Notes

• The payment categories are presented in the descending order of their overall value.

• Grants and donations: A Jonckheere-Terpstra test showed a significant trend in decreasing median payments with increasing years (p <0.001).

• Contributions to costs of events: A Jonckheere-Terpstra test showed no trend in median payments with increasing years (p = 0.767).

• Fees for service and consultancy: A Jonckheere-Terpstra test showed a significant trend in decreasing median payments with increasing years (p <0.001).

• Joint working: A Jonckheere-Terpstra test showed no trend in median payments with increasing years (p = 0.253).

• Pairwise Mann-Whitney U-tests compared 2016, 2017 and 2018 to 2015, with the Bonferroni correction applied to p values.

• P values below 0.05, indicating statistical significance, are highlighted in bold.

Changes in the yearly number and value of payments within each payment category did not follow a clear pattern. Overall, the number of payments increased in all categories, between 8.33% (grants and donations) and 148.18% (contributions to costs of events). Only grants and donations saw an overall decrease in the value of payments, by 27.44%, but the value of the remaining categories increased between 5.14% (joint working) to 61.55% (fees for service and consultancy). Correspondingly, the number of NHS trusts obtaining grants and donations dropped from 184 (85.98%) to 171 (81.43%), while the number of those receiving fees for service and consultancy increased from 82 (38.32%) to 129 (61.43%) (S5 Table in S1 File).

We detected a significant decreasing trend in the median value of payments for grants and donations, which dropped by 60.83%, reflecting the pattern of increasing payment numbers and decreasing value described above. Similarly, a relatively small increase in payment value compared to payment number was associated with significantly decreasing median payments for fees for service and consultancy, by 68.55%. In both payment categories, we found significant differences between median payment values in individual years.

### Foundation and non-foundation NHS trusts

There were more than twice as many foundation trusts receiving pharmaceutical industry payments as non-foundation trusts (Table 3), with the number and value of payments received by foundation trusts higher by 47.25% and 62.94%, respectively, than those made to non-foundation trusts. Nevertheless, both foundation and non-foundation trust had similar median values calculated for the entire period of observation (£230.12 vs £208.33).

**Table 3:** Drug company payments to NHS foundation trusts and non-foundation NHS trusts, 2015–2018.

Trust type	Year	Number of trusts	Value of payments	Number of payments	Mean	Standard deviation	Median yearly payment (IQR)	P-value
**Foundation trusts**	2015	143	£9,812,141.64	4,259	£2,303.86	£10,184.27	£254.40 (£152.64-£551.20)	*Ref*
	2016	148	£12,009,834.42	5,374	£2,234.80	£9,753.17	£209.90 (£125.94-£472.28)	**<0.001**
	2017	142	£11,646,890.17	5,345	£2,179.03	£12,344.68	£212.82 (£139.15-£511.58)	**<0.001**
	2018	138	£10,492,904.11	5,220	£2,010.13	£9,578.84	£240.00 (£150.00-£560.49)	0.424
	All years	158	£43,961,770.35	20,198	£2,176.54	£10,545.87	£230.12 (£144.16-£524.75)	*NA*
**Non-foundation trusts**	2015	71	£4,856,282.52	2,258	£2,150.70	£10,640.94	£254.40 (£148.40-£530.00)	*Ref*
	2016	72	£4,235,976.83	2,826	£1,498.93	£8,611.87	£201.92 (£104.95-£403.01)	**<0.001**
	2017	72	£3,805,127.22	2,894	£1,314.83	£7,334.91	£204.63 (£118.89-£409.27)	**<0.001**
	2018	72	£3,394,264.95	2,677	£1,267.94	£5,976.97	£200.00 (£125.00-£400.00)	**<0.001**
	All years	76	£16,291,651.52	10,655	£1,529.01	£8,206.48	£208.33 (£122.78-£419.80)	*NA*

Notes

• The NHS trusts are presented in the descending order of their overall value.

• Foundation trusts: A Jonckheere-Terpstra test showed no trend in median payments with increasing years (p = 0.161).

• Non-foundation trusts: A Jonckheere-Terpstra test showed a significant trend of decreasing median payments with increasing years (p <0.001).

• Pairwise Mann-Whitney U-tests compared 2016, 2017 and 2018 to 2015, with the Bonferroni correction applied to p values.

• P values below 0.05, indicating statistical significance, are highlighted in bold.

The number of foundation trusts receiving payments decreased by 3.50%. Despite the overall increases of the number and value of payments (by 22.56% and 6.94%), we found no significant trend in median payments. This might reflect the fact that the number and value of payments to these trusts increased by 26.18% and 22.40%, respectively, between 2015 and 2016, but then it kept decreasing.

The number of non-foundation trusts receiving payments stayed almost the same, with the value of payments dropping consistently, resulting in a 30.11% overall decrease. However, the value of payments increased by 18.56%. Consequently, we found a significant decreasing trend in median payment values, with the overall 21.38% reduction. The pairwise comparisons also showed significant differences between payments made in 2015 and in the subsequent years.

### NHS trusts service profiles

Acute trusts constituted the largest group of payment recipients (45.73%), with a similar number of organisations receiving payments each year (Table 4). Acute trusts also accumulated the highest number (68.11%) and value (71.51%) of payments. Save for ambulance trusts, by far the smallest recipient category, NHS trusts with all service profiles had the same median payment value for the entire period of observation (£212.00).

**Table 4:** Drug company payments to NHS trusts with different service profiles, 2015–2018.

	Year	Number of trusts	Value of payments	Number of payments	Mean	Standard deviation	Median yearly payment (IQR)	P-value
**Acute trusts**	2015	104	£10,664,855.46	4,391	£2,428.80	£11,138.45	£254.40 (£159.00-£533.39)	*Ref*
	2016	107	£11,711,755.71	5,517	£2,122.85	£10,311.41	£209.90 (£125.94-£419.80)	**<0.001**
	2017	102	£10,793,356.28	5,637	£1,914.73	£11,812.87	£204.63 (£127.90-£464.11)	**<0.001**
	2018	103	£9,916,019.71	5,469	£1,813.13	£8,835.72	£228.80 (£150.00-£500.00)	**<0.001**
	All years	107	£43,085,987.16	21,014	£2,050.35	£10,565.24	£212.00 (£140.00-£498.15)	*NA*
**Integrated care**	2015	68	£3,657,495.23	1,826	£2,003.01	£9,015.46	£242.21 (£132.50-£530.00)	*Ref*
	2016	70	£4,045,781.76	2,308	£1,752.94	£7,289.92	£209.90 (£124.16-£419.80)	**<0.001**
	2017	70	£4,212,897.15	2,309	£1,824.55	£8,758.65	£204.63 (£122.78-£491.12)	**<0.001**
	2018	65	£3,800,488.56	2,154	£1,764.39	£8,275.73	£240.00 (£144.02-£500.00)	1.000
	All years	72	£15,716,662.71	8,597	£1,828.16	£8,323.40	£212.00 (£126.87-£490.00)	*NA*
**Community**	2015	26	£192,600.94	202	£953.47	£3,192.36	£212.00 (£139.92-£497.51)	*Ref*
	2016	24	£377,116.93	264	£1,428.47	£6,539.32	£218.30(£160.89-£478.36)	1.000
	2017	22	£321,553.46	239	£1,345.41	£5,236.99	£239.01 (£163.71-£491.12)	1.000
	2018	23	£126,840.11	222	£571.35	£1,622.95	£200.00 (£160.00-£400.00)	1.000
	All years	29	£1,018,111.44	927	£1,098.29	£4,706.71	£212.00 (£160.00-£451.00)	*NA*
**Mental health**	2015	14	£152,200.52	95	£1,602.11	£4,076.55	£212.00 (£102.61-£551.20)	*Ref*
	2016	17	£107,630.51	108	£996.58	£2,831.92	£128.46 (£77.24-£419.80)	0.111
	2017	16	£81,524.03	50	£1,630.48	£3,791.61	£368.34 (£162.17-£974.67)	0.835
	2018	18	£43,612.34	51	£855.14	£1,686.89	£240.00 (£160.00-£520.00)	1.000
	All years	19	£384,967.40	304	£1,266.34	£3,300.55	£212.00 (£87.47-£524.75)	*NA*
**Ambulance**	2015	2	£1,272.00	3	£424.00	£367.19	£212.00 (£212.00-£530.00)	*Ref*
	2016	2	£3,526.34	3	£1,175.45	£1,708.73	£188.91 (£188.91-£1,668.71)	*NA*
	2017	4	£42,686.49	4	£10,671.62	£9,580.88	£7,776.06 (£6,169.69-£12,277.99)	*NA*
	2018	1	£208.33	1	£208.33	*NA*	£208.33 (£208.33-£208.33)	*NA*
	All years	7	£47,693.15	11	£4,335.74	£7,314.13	£848.00 (£210.17-£5,257.66)	*NA*

Notes

• The NHS trusts are presented in the descending order of their overall value.

• Due to the small number of payments made to Ambulance trusts, no standard deviation could be calculated for 2018 and no Jonckheere-Terpstra test could be conducted for any years.

• Acute trusts: A Jonckheere-Terpstra test showed a significant trend in decreasing median payments with increasing years (p <0.001).

• Integrated care trusts: A Jonckheere-Terpstra test showed no trend in median payments with increasing years (p = 0.406).

• Community trusts: A Jonckheere-Terpstra showed no trend in median payments with increasing years (p = 0.129).

• Mental health trusts: A Jonckheere-Terpstra test showed no trend in median payments with increasing years (p = 0.924).

• Ambulance trusts: A Jonckheere-Terpstra test showed no trend in median payments with increasing years (p = 0.883).

• Pairwise Mann-Whitney U-tests compared 2016, 2017 and 2018 to 2015, with the Bonferroni correction applied to p values. The Mann-Whitney U-test could not be computed for payments to ambulance trusts due to the low number of payments to this type of trust.

• P values below 0.05, indicating statistical significance, are highlighted in bold.

The yearly number and value of payments fluctuated, but only integrated care trusts saw an overall increase of the value of payments. Conversely, the number of payments in all trust types except for mental health and ambulance trusts saw an overall increase. Considering the median payment values, we only observed a significant decreasing trend in acute trusts (p <0.001), with the median dropping by 10.06%.

### Regions of England

North-West England has the highest count of trusts receiving payments, with 42 trusts making up 17.95% of the total (Table 5). London, while having fewer trusts receiving payments (39 in total, or 16.67% of all trusts), leads in terms of payment values and quantity. London’s trusts received the highest proportion of payment value (30.78%) and the greatest number of payments (16.25%), despite not having the most trusts.

**Table 5:** Drug company in payments to NHS trusts across the regions of England, 2015–2018.

Region	Year	Number of trusts	Value of payments	Number of payments	Mean	Standard deviation	Median yearly payment (IQR)	P-value
**Greater London**	2015	36	£3,858,711.00	1032	£3,739.06	£3,739.06	£339.20 (£152.64–£1590.00)	*Ref*
	2016	37	£4,652,404.02	1325	£3,511.25	£3,511.25	£251.88 (£100.75–£997.03)	**<0.001**
	2017	37	£5,387,014.95	1341	£4,017.16	£4,017.16	£235.74 (£91.68–£1023.17)	**<0.001**
	2018	38	£4,647,100.25	1316	£3,531.23	£3,531.23	£300.00 (£150.00–£1200.00)	0.051
	All years	39	£18,545,230.22	5014	£3,698.69	£3,698.69	£262.38 (£120.00–£1200.00)	*NA*
**North West**	2015	40	£2,109,864.92	881	£2,394.85	£2,394.85	£282.38 (£159.00–£750.48)	*Ref*
	2016	39	£2,598,880.50	1038	£2,503.74	£2,503.74	£251.88 (£126.99–£524.75)	**<0.001**
	2017	39	£2,804,119.91	1035	£2,709.29	£2,709.29	£245.56 (£127.69–£624.03)	**0.008**
	2018	37	£2,141,443.83	962	£2,226.03	£2,226.03	£320.00 (£148.00–£717.40)	1.000
	All years	42	£9,654,309.16	3916	£2,465.35	£2,465.35	£264.00 (£136.01–£631.28)	*NA*
**South East**	2015	26	£1,763,804.81	1095	£1,610.78	£1,610.78	£212.00 (£152.64–£424.00)	*Ref*
	2016	27	£2,224,900.96	1346	£1,652.97	£1,652.97	£209.90 (£130.98–£393.56)	**0.001**
	2017	25	£1,393,178.46	1303	£1,069.21	£1,069.21	£204.63 (£133.01–£409.27)	**0.017**
	2018	26	£1,589,470.98	1381	£1,150.96	£1,150.96	£201.23 (£152.00–£384.00)	0.401
	All years	27	£6,971,355.21	5125	£1,360.26	£1,360.26	£209.90 (£143.24–£400.00)	*NA*
**North East**	2015	22	£1,457,330.82	616	£2,365.80	£2,365.80	£296.80 (£159.32–£821.18)	*Ref*
	2016	22	£1,394,690.91	805	£1,732.54	£1,732.54	£218.30 (£125.94–£503.76)	**<0.001**
	2017	22	£1,865,526.74	788	£2,367.42	£2,367.42	£255.79 (£148.36–£615.95)	0.089
	2018	22	£1,168,801.77	747	£1,564.66	£1,564.66	£248.04 (£150.00–£520.00)	**0.007**
	All years	24	£5,886,350.24	2956	£1,991.32	£1,991.32	£255.79 (£143.83–£592.34)	*NA*
**South West**	2015	23	£1,483,270.30	784	£1,891.93	£1,891.93	£233.20 (£159.00–£424.00)	*Ref*
	2016	23	£1,861,086.71	975	£1,908.81	£1,908.81	£209.90 (£125.94–£348.86)	**<0.001**
	2017	24	£936,373.51	1016	£921.63	£921.63	£204.63 (£122.78–£375.76)	**<0.001**
	2018	23	£1,000,513.47	892	£1,121.65	£1,121.65	£208.33 (£150.00–£400.00)	**0.007**
	All years	25	£5,281,243.99	3667	£1,440.21	£1,440.21	£209.90 (£132.00–£400.00)	*NA*
**West Midlands**	2015	26	£1,671,701.83	849	£1,969.02	£1,969.02	£254.40 (£159.00–£508.80)	*Ref*
	2016	27	£1,415,580.25	1092	£1,296.32	£1,296.32	£209.90 (£125.94–£419.80)	**<0.001**
	2017	24	£994,792.20	1003	£991.82	£991.82	£204.63 (£127.38–£409.27)	**<0.001**
	2018	25	£1,219,393.34	982	£1,241.74	£1,241.74	£208.33 (£140.00–£450.00)	**0.024**
	All years	30	£5,301,467.62	3926	£1,350.35	£1,350.35	£209.90 (£132.53–£424.00)	*NA*
**East Midlands**	2015	19	£1,058,143.30	667	£1,586.42	£1,586.42	£186.56 (£148.40–£424.00)	*Ref*
	2016	21	£963,732.06	764	£1,261.43	£1,261.43	£179.68 (£117.54–£314.85)	**0.001**
	2017	21	£1,096,367.41	921	£1,190.41	£1,190.41	£163.71 (£135.06–£327.41)	**0.001**
	2018	17	£1,278,832.03	770	£1,660.82	£1,660.82	£179.50 (£140.00–£360.00)	0.676
	All years	22	£4,397,074.81	3122	£1,408.42	£1,408.42	£176.56 (£137.02–£355.95)	*NA*
**East England**	2015	22	£1,265,597.18	593	£2,134.23	£2,134.23	£212.00 (£139.92–£424.00)	*Ref*
	2016	24	£1,134,535.84	855	£1,326.94	£1,326.94	£201.50 (£114.40–£335.84)	**0.001**
	2017	22	£974,644.22	832	£1,171.45	£1,171.45	£204.63 (£146.31–£409.27)	1.000
	2018	22	£841,613.39	847	£993.64	£993.64	£200.00 (£150.00–£400.00)	1.000
	All years	25	£4,216,390.62	3127	£1,348.38	£1,348.38	£204.63 (£144.00–£400.19)	*NA*

Notes

• The NHS trusts are presented in the descending order of their overall value.

• London: A Jonckheere-Terpstra test showed a significant trend in decreasing median payments with increasing years (p = 0.005)

• North West England: A Jonckheere-Terpstra test showed no trend in median payments with increasing years (p = 0.525)

• South East England: A Jonckheere-Terpstra test showed a significant trend in decreasing in median payments with increasing years (p = 0.039)

• North East England: A Jonckheere-Terpstra test showed a significant trend in decreasing median payments with increasing years (p = 0.013)

• South West England: A Jonckheere-Terpstra showed a significant trend in decreasing median payments with increasing years (p = 0.005)

• West Midlands: A Jonckheere-Terpstra test showed a significant trend in decreasing median payments with increasing years (p = 0.003í)

• East Midlands: A Jonckheere-Terpstra test showed no trend in n median payments with increasing years (p = 0.089)

• East England: A Jonckheere-Terpstra test showed no trend in median payments with increasing years (p = 0.883)

• Pairwise Mann-Whitney U-tests compared 2016, 2017 and 2018 to 2015, with the Bonferroni correction applied to p values.

• P values below 0.05, indicating statistical significance, are highlighted in bold.

An overall increase in the number of payments was observed in all regions, between 9.19% (North West of England) and 42.83% (East England). However, the growth occurred only in the first year or two and was followed by subsequent falls. Between 2015 and 2018 the value of payments increased only in North West England (1.50%), London (20.43%), and East Midlands (20.86%). The annual value of payments fluctuated, with only East England seeing a steady decrease.

A trend towards decreasing median payments was observed in all regions except East Midlands, with the medians dropping between 5.56% (London) and 53.44% (East England). In two of the eight regions significant differences existed in median payments made in 2015 and the three subsequent years.

### Explanatory models

Table 6 presents the random-effects (Models 1,2,3, and 4) explanatory models with the log transformed sum *value* of payments per trust per year as the outcome. Model 1 had trust type as the sole explanatory variable, and then further variables were added incrementally to models 2, 3, and 4 to check whether any additional variable changed the significance and/or the coefficient of other variables drastically. Save for South East England, which lost its significance in comparison to the reference category of London, all other variables’ significance and coefficients remained stable when other variables were added to the model.

**Table 6:** Random effects explanatory models on payment values per NHS trusts per year.

	Model 1	Model 2	Model 3	Model 4
**Mental health trusts**	-3.552** (-0.591)	-3.545** (-0.59)	-3.598** (-0.58)	-3.598** (-0.581)
**Ambulance trusts**	-7.652** (-0.927)	-7.685** (-0.926)	-7.687** (-0.911)	-7.687** (-0.913)
**Community health trusts**	-3.529** (-0.497)	-3.544** (-0.497)	-3.429** (-0.486)	-3.429** (-0.487)
**Integrated health trusts**	-1.274** (-0.362)	-1.253** (-0.362)	-1.319** (-0.359)	-1.319** (-0.36)
**Foundation trusts**		-0.36 (-0.332)	-0.36 (-0.33)	-0.36 (-0.331)
**East England**			-3.419** (-1.219)	-3.419** (-1.221)
**East Midlands**			-1.025 (-0.619)	-1.025 (-0.62)
**East of England**			-0.003 (-0.629)	-0.003 (-0.63)
**North East**			-0.176 (-0.609)	-0.176 (-0.61)
**North West**			-0.379 (-0.52)	-0.379 (-0.521)
**South East**			0.383 (-0.58)	0.383 (-0.581)
**South West**			0.106 (-0.596)	0.106 (-0.597)
**West Midlands**			-0.918 (-0.563)	-0.918 (-0.564)
**2016**				0.209 (-0.164)
**2017**				-0.003 (-0.164)
**2018**				-0.326* (-0.164)
**Constant**	10.081** (-0.23)	10.320** (-0.318)	10.633** (-0.44)	10.663** (-0.452)
**Observations**	936	936	936	936
**R2**	0.118	0.12	0.14	0.148
**Adjusted R2**	0.114	0.115	0.127	0.133
**F Statistic**	124.585***	126.258***	149.565***	159.919***

Note: Significance levels are

*p<0.05;

**p<0.01.

Reference categories are acute trusts, non-foundation trusts, London, and 2015.

P values below 0.05, indicating statistical significance, are typed in bold.

The table was formatted using the R stargazer package [79]

The final model (Model 4) shows acute trusts received a significantly higher value of payments than all other trust types when other variables were held constant; this difference ranged between 73.25% compared to integrated health trusts and 99.95% more value of payments in comparison to ambulance trusts on average. There was also a significant difference in the value of payments between trusts based in London and in East England, the latter receiving 96.73% less on average. Considering changes over years, payment values in 2018 were significantly different from 2015, 27.80% less on average, when all other variables were held constant. Importantly, foundation NHS trusts did not receive significantly higher value of payments than non-foundation trusts. The final regression model explained 13.3% of the variance (adjusted R^2^ = 0.133) in the data (F = 159.92, p < 0.01).

The explanatory model of the effects of trust characteristics on the *number* of payments showed that acute trusts received significantly (p<0.01) more payments than all other trust types, between 52.40% and 96.66% more in the case of integrated health trusts and ambulance trusts, respectively (S6 Table in S1 File). Trusts in South East England received 70.23% (p<0.01) more payments than trusts in London, and all trust types received 16.67% (p<0.01) more payments in 2016 compared to 2015. The random-effects model with all explanatory variables explained 15.8% of the variance (adjusted R^2^ = 0.158) in the data (F = 191.99, p < 0.01).

## Discussion

We conducted the first longitudinal analysis of payments to HCOs in a European country with self-regulation of payment disclosure by the pharmaceutical industry. We now summarise and interpret the key patterns in financial ties between drug companies and English NHS trusts. We then highlight the study’s limitations. We then outline the study’s implications for the understanding of institutional COIs and draw out our policy recommendations.

### Key patterns in drug company payments to NHS trusts

Our findings suggest that the ties between drug companies and NHS trusts were widespread and stable. More than two-thirds of companies which subscribed to the pharmaceutical industry’s self-regulatory rules made non-R&D payment to NHS trusts at least once, and more than a half did so consistently between 2015 and 2018 [36]. Pharmaceutical industry payments were prevalent among NHS trusts, too, with around nine out of ten receiving them in any given year, and eight out of ten consistently throughout the period of observation. Compared with an earlier study of English general practices, the number of companies making payments to NHS trusts in 2015 was 2.2 times higher (83 vs 37), the number of payments also 2.2 higher (6.5k vs 2.9k), and the value of payments 5.4 times higher (£14.7m vs £2.7m) [36]. The dominance of NHS trusts over general practices is unsurprising as most new medicines, including high-cost specialist therapies, are prescribed in hospitals [80–82].

Contrastingly, we also detected the decreasing value of non-R&D payments to NHS trusts as a share of payments to HCOs. One possible hypothesis explaining this pattern is the diminishing importance of NHS trusts as a payment target relative to other HCOs. Yet, as Disclosure UK lacks built-in recipient categories it is impossible to establish—without extensive forensic research involving categorisation of all payment recipients—where the pharmaceutical industry’s focus has shifted [26]. An alternative explanation would point not to the decreasing importance of NHS trusts as such but only grants and donations as the dominant payment category in terms of value, which inevitably affected the total value of payments to NHS trusts. This explanation is made plausible by the overall increase in the number and value of all the payment categories except for grants and donations. It is further reinforced by the stable number and share of trusts receiving payments annually as well as the increase in the number of companies reporting payments to NHS trusts.

A complementary explanation might be that the non-R&D payments, especially grants and donations, were overtaken by R&D payments, which constitute around two-thirds of all payments reported by drug companies in the UK [83]. The ABPI Code allows for R&D payments to be reported without mentioning any organisational- or individual-level recipients [83]. NHS trusts are likely to receive a substantial share of R&D payments, including for research and infrastructure, consultancy fees for clinical trials, and support for attendance at project meetings and conferences [83]. Indeed, many NHS trusts are listed as research sites in commercial clinical trials [84], with the NHS receiving £350m from commercial research sponsors in 2019 alone, which dwarfs the value of the non-R&D payments we analysed [85]. An increase in the value of R&D payments relative to other payment types would also correspond with the growing policy emphasis on “embedding” clinical research delivery in the NHS, which involves encouraging commercial research within the NHS by “ensur[ing] faster approval, set-up and delivery of studies with more predictability and less variation” [86].

We hypothesise a two-fold change in drug company payment strategies vis-à-vis NHS trusts. First is the move from grants and donations to fees for service and consultancy and contributions to costs of events, indicated by the contrasting changes in the number of recipients as well as the number and value of payments. Without contextual information on the nature of the funded projects and activities it is difficult to explain this potential shift. Second, the significant trends in decreasing median payment values overall as well as in relation to NHS trusts with specific governance, service, and geographical profiles. The decreasing median payment values may indicate a move towards a strategy relying on smaller but more frequent payments which was observed in a subset of general practices in England [36] and documented extensively in payments to physicians made in the US [87–89]. What indicates the move towards the “small payments” strategy in relation to NHS trusts is the fact that the drop in the overall value of payments (5.33%) was four times lower than the increase in their number (21.18%). This interpretation is also consistent with the diminishing importance of grants and donations (involving less frequent but high-value payments) and the increasing importance of fees for services and consultancy, and especially contributions to costs of events (frequent but lower-value payments).

We can speculate that using small payments may simply be a more cost-effective strategy of engaging with NHS trusts. For example, in the US, small payments, especially for food and beverages, have been associated with significant increases in prescription of marketed products [4, 87, 90]. This may reflect their role in building trust-based social relations with physicians and other staff seen as vital in generating additional prescriptions [91–93]. It is possible that frequent small payments to NHS trusts are involved in similar promotional strategies, involving regular, confidence-inducing presence, which seek to influence organisational processes. Another possible explanation is the growing publicity given to the disclosure requirements and their implementation, including examples high-level press and TV coverage [94–96]. This increased media and policy attention may result in donors and recipients seeking to avoid larger payments as they carry higher reputational risks [97, 98]. This interpretation would be consistent with joint working—the only payment category associated with expectations regarding contextual information about funded projects—being associated with higher-value payments.

The example of joint working also underscores that the shift to small payments was not uniform. Indeed, this payment category consistently represented the opposite “high-payments” strategy, associated with providing extensive payments for a limited number of projects, which was also observed when analysing payments to all HCO types across the UK [26]. The importance of this strategy, also pursued by some drug companies in relation to general practices in England [36], is underscored by evidence of a dose-response relationship between payments and physician prescribing identified in the US [99, 100].

Consistent with the identified trends in payments to NHS trusts, our final random effects models indicated that the total value—but not the number—of payments received by NHS trusts were significantly lower on average in 2018 than those received in 2015. Conversely, the models suggest that throughout the period of observation, the pharmaceutical industry prioritised making payments to acute trusts, the NHS trusts focused on delivering hospital care and accident and emergency services and that generate the lion’s share of prescription costs of hospital drugs in England, including many new, costly drugs [101]. Furthermore, many acute trusts are teaching hospitals or have ties to universities [46], which may provide another reason for companies to prioritise them. Contrasting with London receiving the highest healthcare spend per person in England [102], drug companies prioritised NHS trusts based in London only when compared with those from East England. In addition, while the higher autonomy from central government—and therefore more potential for collaborations with the pharmaceutical industry—might have made foundation trusts a more attractive payment recipient, they did not receive a significantly higher value of payments than non-foundation trusts. This finding is surprising and warrants further investigation, especially in light of new evidence from the US suggesting that pharmaceutical industry payments to healthcare professionals may be related to their level of clinical autonomy, including in prescribing decisions [103, 104].

As the final models explained only 13.3% and 15.8% of the variance of the value and number of payments, other important variables are also behind the payment patterns. Notably, US research suggest that hospital size, affiliation with major medical schools, and quality of services were significant predictors of receiving payments by teaching hospitals [21]. Similarly, what suggests the importance of hospital department profiles (e.g. cardiology, oncology) is considerable differences in payment values to different medical specialties [105–107] and subspecialties [108, 109]. Nevertheless, many potentially relevant NHS trust characteristics are difficult to collect systematically, given the substantial differences in the content of annual reports published by NHS trusts, while others, such as data on prescription of hospital drugs, are not publicly available [80].

### Limitations

As we took a conservative approach to data extraction, we only report a *minimum* number and value of payments to NHS trusts [110]. We extracted payments using forensic qualitative searches triangulating different pieces of information. Nevertheless, it is possible that some payments were missed due to errors in the naming of NHS trusts by drug companies of which we were unaware. However, we expect the share of these payments to be negligible given a comprehensive combination of search terms, supplemented by postcode searches.

Another important limitation is the short time series spanning only four years, resulting from an excessive amount of work involved in data extraction and cleaning. In combination with the yearly fluctuations in the number and value of payments, it constrains our ability to draw definitive conclusions about the strength of the effects we observed. The lack of inclusion of more recent years is a related key limitation, although including data from the unique Covid-19 years could also be problematic in terms of generalising the results. Specifically, high-level data summaries published by the ABPI indicate that the effects of the Covid-19 pandemic on payments could be mixed: while the overall value of non-R&D payments to healthcare professionals and organisations dropped from 160.9m to 138.9m between 2019 and 2020, and then recovered somewhat to 152m in 2021 [111, 112], the value of grants and donations to HCOs also saw considerable fluctuation—a surprising increase from 44.2m to 53.0m, followed by a drop to the pre-pandemic levels in 2021 (44.0m) [111, 112].

Finally, the narrow set of the analysed NHS trust characteristics, which results from insufficient data being publicly available in a standardised format, excluded potentially important explanatory variables.

### Implications for governance of institutional conflicts of interest

As drug company payments are made for “promotional purposes or otherwise, in connection with the development or sale of [prescription] medicines” (ABPI Code, Clause 1.10, 24.1–2) [64], they may generate institutional COIs. Their nature, extent, and consequences in hospital settings have only recently been gaining prominence as a subject of inquiry, albeit in countries with health systems much different from the NHS in England. For instance, an analysis of the distribution of payments to US teaching hospitals highlighted concerns about institutional COIs undermining patient care, research, medical education and training, as well as hospital spending [21]. Beyond the US, an investigation into institutional COIs involving a Japanese teaching hospital underscored, among others, risks to patient care, research and stewardship of hospital resources [113]. In addition, further evidence exists of institutional COIs being involved in obfuscating a drug company’s role in sponsoring a clinical trial in Japan [114].

Nevertheless, many institutional COIs arising as a result of accepting drug company payments by NHS trusts may go unnoticed, and so is their possible impact on clinical and organisational decision-making (e.g. prescription patterns, procurement, investment). Examining potential institutional COIs using Disclosure UK is difficult without contextual information on grants and donations made by companies, other than they may take the form of “medical and educational goods and services”, such as patient therapy review or screening services or support for investigator-led trials [115]. Similarly, it is unclear how contributions to costs of events are utilised, apart from the provision that they can sponsor meetings held by NHS trusts or staff participation at external events. Further, no detail is available of consultancies or other services rendered by NHS trusts to drug companies. Only in relation to joint working, constituting the smallest and second-smallest payment category in terms of the number and value of payments, respectively, are companies expected to provide a web link with a project description [64].

Another blind spot is payments associated with payments made to NHS trusts by medical device companies, which may be especially relevant for hospitals as they are major purchasers, leasers and users of medical technologies, both for their own use (e.g., laboratory equipment) and patients’ [116]. For example, medical device companies have been listed as major donors to teaching hospitals in the US [21, 117]. Contrastingly, in the UK, medical device companies reported their payments under separate self-regulatory arrangements, only covering “contributions to medical education” [118].

These gaps in the industry self-regulation are not addressed adequately by the current COI guidance for NHS trusts [119, 120], which focuses on individual staff. Institutional COIs, although not defined explicitly as such, are discussed in relation to three forms of financial relationships with external partners, and only partially overlap with the payment categories from the pharmaceutical industry’s self-regulation. First, sponsorship of events can only be accepted by NHS trusts if it “will result in clear benefit for the organisation and the NHS”, but it is not made clear how the benefits are operationalised and evaluated. In addition, the acceptance of such sponsorship must not result in breaching the confidentiality of personal data, gaining undue competitive advantage, or shaping the event’s purpose [119]. Second, the guidelines stipulate that research sponsorship “must not constitute an inducement to prescribe, supply, administer, recommend, buy or sell any medicine, medical device, equipment or service” [119]. Third, sponsored posts, which are sometimes part of joint working projects with drug companies [38], “should not have any undue influence over the duties of the post or have any preferential access to services, materials or intellectual property relating to or developed in connection with the sponsored posts” [119]. Finally, the guidelines mention the possibility of accepting gifts (i.e. cash, goods, services) worth over £50, and donations (i.e. charitable payments) “on behalf of an organisation”.

The implementation of the current-sector wide COI guidance by individual trusts, and how it might have been affected by their institutional cultures and policies, has not been investigated in relation to institutional COIs. However, compliance with the guidelines in relation to publishing individual-level COI declarations has been described as poor [121] or not always consistent across the studied trusts [122]. In addition, gaps in the reporting of joint working projects with drug companies suggest that at least some trusts lack a complete picture of their financial ties with external organisations [38]. Similarly, the underreporting of payments by NHS clinical commissioning groups indicates that not all payments made by drug companies are interpreted as falling under the official COI guidelines to be followed by NHS organisations [37]. These deficiencies in enforcement are compounded by the dispersal of disclosures on hundreds of trust websites, their electronic format (typically, PDFs) hindering data searchability and integration, as well as disparate approaches to data presentation [121].

Another key area for improved governance is the risk of excessive reliance on pharmaceutical industry payments, underscored by the rapidly growing demand for health services combined with insufficient central funding, resulting in, for example, falling bed capacity and increasing waiting lists [123, 124]. Therefore, some NHS trusts, their parts, or specific budget lines, for example, for travel and events, may to a large extent draw on drug company income. Similarly, providing consultancy and other services to drug companies may be an increasingly attractive source of alternative income. Although the effects of financial dependency are difficult to measure, there is near-consensus that it may introduce commercial bias to organisational agendas and cultures [110, 125]. Consequently, we need a better understanding of the small subset of NHS trusts in relation to which drug companies reported no payments. It would be important to ascertain whether these organisations were not considered suitable payment targets (and, if so, why) or whether they did not accept payments that had been offered (and to what extent this decision was affected by their organisational cultures and policies).

### Policy recommendations

Our policy recommendations fall into three principal areas: (1) disclosure requirements within the pharmaceutical industry’s self-regulation; (2) institutional COI guidance and education within the NHS; and (3) arrangements for governing collaborations with the health service by drug companies. While the focus of our recommendations is on England, their principles are applicable to other countries with publicly funded health systems with transparency of collaborations with drug companies governed through a mixture of industry self-regulation and regulations developed by their public-sector partners.

Improving the quality of reporting in Disclosure UK is vital as it is being considered as a possible foundation for future regulation of payment disclosure [126, 127]. Our study supports arguments for enhancing its information content, including individualising R&D payments [83] and providing information on the marketed product(s) relevant to each payment, following the example of the US Open Payments database [24, 26]. As argued before, introducing mandatory recipient identifiers is crucial for efficient analysis and making the disclosure process more accountable to the public [24]. This would allow, for example, establishing unambiguous connections between payments to healthcare professionals and their employers, drawing on the example of individual- and organisational-level identifiers used in the Open Payments database. As a rule, payments should be reported at the lowest level of aggregation (e.g. hospital clinic or department), while providing the name of the main recipient (e.g. NHS trust). It is therefore imperative to report payments made to organisational subunits, which should involve clarifying the meaning of the “location” column in Disclosure UK and making it mandatory. In addition, introducing a recipient categorisation is necessary to gain insights into the distribution of payments in the health system and over time. Indeed, the database should take the advantage of publicly available list of potential recipients, such as NHS trusts, general practices or clinical commissioning bodies, and use them as the source of organisation names. Introducing meaningful payment descriptions is also necessary and consistent with calls for more contextual information that were raised regarding payments made to teaching hospitals in the US [23]. Finally, detecting trends in payments requires keeping Disclosure UK datasets in the public domain permanently, as opposed the current minimum three-year data retention period [64].

The COI guidance for NHS organisations needs to engage comprehensively with the notion of institutional COIs. This would reflect the fact that some NHS organisations seem to “outsource”—implicitly or explicitly—financial disclosures to Disclosure UK [37, 38], which was not designed to cover all information relevant for the public, including decisions at stake or the likely bias the payments may introduce [128]. The need for an updated comprehensive NHS guidance is also made evident by the very limited disclosure levels by medical device manufacturers, which may not capture institutional COIs which are key in the context of services provided by NHS trusts. Any revisions of the guidance should prioritise putting the COI disclosure standards in line with those routinely applied to inspections of the safety and quality of care [129] or patient satisfaction surveys [130], including creating a centralised COI database. Separately, more attention is needed to COI management, including whether, and, if so, to what extent and to what purpose corporate grants, donations, conferences sponsorships, consultancies or collaborations are acceptable for publicly funded NHS organisations. This would be key to addressing the risks of “strategic exaggeration” and “moral licensing” at the organisational level [131]. Both the disclosure and management of institutional COIs should become part of induction and training for organisational managers and leaders within the NHS.

Finally, other key areas for policy discussion involve setting acceptable levels of external payments within NHS organisations, including specific budgets and individual activities. One possible way of addressing this issue, put forward in relation to drug company payments to patient organisations [132], would be a single pool of funding available to all NHS organisations.

## Supporting information

S1 File (DOCX)

S1 Data (DOCX)
