# Supplementary material for: Pharmaceutical industry payments to NHS trusts in England: A four-year analysis of the Disclosure UK database
Source: PLoS One. 2023 Nov 1;18(11):e0290022. doi: 10.1371/journal.pone.0290022 (PMC10619808; doi:10.1371/journal.pone.0290022)
Supplement: S1 File — (DOCX) [file pone.0290022.s001.docx]

# Appendices

## S1 Appendix Data extraction and management protocol

We used three data sources: (1) Disclosure UK; (2) methodological notes submitted by drug companies reporting payments in Disclosure UK; and (3) NHS trust annual accounts. In what follows below, we characterise the process of data extraction, cleaning, and organisation applied to each of these three data sources.

### 1. Disclosure UK databases

#### Databases used

We considered four yearly versions of Disclosure UK from 2015-18 (Excel file names: Disclosure UK 2015 (ABPI Full Report 2015 NHS trusts - 2016-06-12), Disclosure UK 2016 (ABPI Full Report 2016 NHS trusts - 2019-06-12), Disclosure UK 2017 (ABPI Full Report 2017) NHS trusts, Disclosure UK 2018 (ABPI Full Report 2018) NHS trusts). We joined the four datasets together into one big dataset but kept the original row numbers for each year to ensure the cleaned and standardised dataset could be matched with the original data based on the year and the row number.

#### Data extraction and cleaning

Payment recipients in Disclosure UK are identified using their names (Name column), locations (Location column), addresses (columns with the first and second line of address), and postcodes. While the expected content of the location column is not formally specified (1), it typically consists further detail on organisations identified using specific names. In practice, the ‘Institution name’ includes organisation names, such as NHS trusts, CCGs, hospitals, pharmacies, companies, universities, charities, societies. These organisations are usually aggregated at a higher level, for example, NHS trusts as opposed to their constituent hospitals. The ‘Location’ column usually involves the name of a specific building, department, hospital of the organisation noted in the organisation name. For example, if the ‘Institution Name’ column denotes the name of an NHS trust, then the ‘Location’ column often provides the hospital belonging to the trust. As noted above, the reporting is not uniform across drug companies due to the lack of specification of the contents of the ‘Location’ column and its relationship to the ‘Institution Name’ column.

Consistent with earlier research on the distribution of drug company payments in a single year (1), extracting and cleaning data on payments to NHS trusts required forensic work. We specify the rationale for undertaking this work below.

1. **Reasons for data cleaning and reorganising the original Disclosure UK dataset**
   1. the study is focused on drug company payments to NHS trusts only but Disclosure UK includes payments to a much broader range of healthcare organisations, such as clinical commissioning groups (CCGs) and private healthcare providers (1), and therefore payments to NHS trusts had to be identified and isolated from the main dataset.
   2. To allow for establishing which NHS trusts received payments as well as calculating the total number of recipients, the NHS trust names had to be standardised (1). Name standarisation was necessary because frequently the name of an NHS trust was typed differently by different drug companies or even the same company within a single year and particularly over time, for example *James Paget Univ Hosps NHS FT* and *James Paget University Hospitals NHS Foundation Trust* are the same organisation
      1. The standardised NHS trust names come from an official list of NHS trusts (2) in July-August, 2019.
   3. in many instances, the trust name and address details provided in Disclosure UK did not match the address available from the official NHS website, suggesting that the payment recipient was misidentified (i.e. that, in fact, it was not an NHS trust).
   4. there were 21 trusts which merged with other trusts during the study period of observation.
      1. In these instances, we kept the original name of the trust in years before the merger while noting that this trust merged with another in a given year
   5. some addresses associated with payments reported in Disclosure UK related to a location outside an NHS trust premises, such as shared business services. We identified and noted these instances. A key example was addresses related to “NHS Shared Business Services”.
      1. “NHS Shared Business Services provides innovative finance and accounting, employment, procurement and innovation & technology solutions to the NHS.” (NHS Shared Business Services website: <https://www.sbs.nhs.uk>)
      2. We excluded payments to locations which our checks revealed were not related to NHS trusts.

We identified the NHS trusts receiving pharmaceutical industry payments by undertaking the following steps.

1. **First round of identification of NHS trusts**

This round of searches focused on the column: **“ Standardised NHS trust name” – searches used search terms from trust names”**. We created a separate Excel spreadsheet with a list of all standardised trust names obtained from the offical NHS website (3). From each standardised name we derived one, or, in some cases two, search terms that were subsequently used to successfully conduct searches in the Disclosure UK dataset. In Disclosure UK we inserted an additinoal column with standardised trust names to identify payments made to NHS trusts which could be identified based on these search terms. We inserted the standardised trust name to this column ONLY if the trust name could be evidently identified based on searches in the ’Institution name’ column. For example, to identify payments made to *Aintree University Hospital NHS Foundation Trust*, we used the search term “ Aintree”.

- 1. We used the search terms in the Name column. For all results that could be clearly linked with the standardised name of the trust we supplied the standardised name in the separate Standardised NHS trust name column. For example, the standardised trust name of *James Paget Univ Hosps NHS FT* is *James Paget University Hospitals NHS Foundation Trust*

1. **Second round of identification of NHS trusts**
   1. This round of searches focused on the column: **“Standardised NHS trust name – search based on the search term ’hosp’”**
   2. Based on insights from our previous research (1), we anticipated that some NHS trusts would be identified at a lower level of aggregation, that is, by the names of their constituent hospitals. Therefore, we used “hosp” as a generic search term for the second round of searches.
   3. The search term ’hosp’ is used for the “ Institution Name” column in Disclosure UK.
   4. The hospital names identified based on this approach, were entered, together with the associated postcode from Disclosure UK, into the Google search engine.
   5. The online search results were compared with the data regarding specific trusts and hospitals provided by NHS England (4).
   6. Specifically, this website includes the names of NHS trusts as well as associated hospitals, clinics or surgeries. When a hospital could be clearly matched with a specific NHS trust then the standardised name of the trust was supplied in the “Standardised NHS trust name (2nd column)”.
   7. In addition, even if the hospital found in Disclosure UK was not included on the NHS England website, we linked it to an NHS trust from the website as long as the hospital’s postcode from Disclosure UK matched the NHS trust postcode from the official website
      1. There were several cases when the hospital’s name did not appear on the central NHS website of all trusts (2), but the hospital did appear on the website of the NHS trust.
   8. There were several hospitals which could be matched with two different trusts but in these cases they were matched with the one that was given in the Disclosure UK dataset.
      1. e.g. Mount Vernon Hospital (HA6 2RN) appears on the website of both East and North Hertfordshire NHS Trust and The Hillingdon Hospitals NHS Foundation Trust but in the dataset The Hillingdon Hospitals Trust was given so that trust was added in the standarised column.
   9. When a hospital was shown as related to a clinical commissioning group (CCG) based on the website listing NHS hospitals (4), additional websearch was undertaken to eliminate the possibility of the hospital being connected to a trust.
      1. On the NHS website all hospitals/clinics/surgeries of each NHS trust and CCGs are given (4). That is how the trust to which hospitals belong were checked. There were 169 cases, when after a ’hosp’ search in the **’Location’ column** a CCG was given in the ’Institution Name’ column. Following a web search of the hospitals’ name and addresses it was evident that they in fact belonged to an NHS trust, and not a CCG. We put down the name of this trust in the ’Standardised NHS trust name - based on location’ column. These cases can be easily identified by searching for ’CCG’ in the first ’Institution Name’ column and then checking any inputs in the’ Standardised NHS trust name - based on location’ column.
   10. The standardised trust name for the given hospital is inserted here, in the second column
2. **Third round of identification of NHS trusts**
   1. This round of searches also focused on the column: **“ Standardised NHS trust name – search based on the search term ‘hosp’”**
   2. The same column and method was used again for the standardised trust names but this time the search term “hosp” is used for the *Location* column
   3. The hospital names identified through the searches, were looked up in Google together with the given postcode in the dataset.
   4. These searches accounted for the instances in which the hospital name was provided in the location column, while the name column included the name of a department, association, network, society, research group, foundation, etc.
3. **Fourth round of identification of NHS trusts**
   1. This round of searches also focused on the column: “Standardised NHS trust name – search based on the search term “hosp”
   2. The same column and method was used again for the standardised trust names but this time the search term “hosp” is used for the *Address Line 1* column. Once this term has been applied in the Address line column, all payments associated with standardised trust names obtained through the previous searches should be filtered out (that is those from the “Standardsied NHS trust name - searches based on search terms from trust names” column, the **“Standardised NHS trust name - search based on search term ‘hosp’”** and **“Standardised NHS trust name - based on location” column”**), leaving only blanks in the columns with standardised trust names
   3. This round of searches was only used to identify any results that might have been missed in the previous rounds of searches.
   4. The first step was to look at the results from the “Institution name” column (in combination with postcodes) and consider if any of them could be meaningfully linked to NHS trusts.
   5. The next step was to look at the Location column (in combination with postcodes) and consider if any of them could be meaningfully linked to NHS trusts.
   6. For the two steps above we ignored results associated with payments outside England (e.g. in Scotland), medical / scientific societies (we assumed these societies were only using trust premises and as such they were the ultimate recipients of payments), private hospitals and institutions.
4. **Adding standardised postcodes to the standardised trust names**
   1. Given that (1) the Disclosure UK dataset comprises data that were provided by different drug companies in different years; and (2) that we have found several instances when the given trust name and the address did not match, we found it important to check whether the postcodes provided in Disclosure UK indeed belonged to the NHS trusts mentioned in the dataset. To do this, we collected the main official postcode of each trust because presumably those were used the most in the dataset. Any other postcodes had to be checked individually.
   2. standardised postcodes were taken from the NHS website on NHS trusts (<https://www.nhs.uk/servicedirectories/pages/nhstrustlisting.aspx>)
   3. each standardised trust name was selected one by one in the Standardised NHS trust name columns (E-K) and the standardised postcode for the selected trust was inserted in the standardised trust postcode column
   4. in case the postcode provided in Disclosure UK and the standardised postcodes were different, additional websearch was needed to ensure the payment recipient institution belonged to the trust
   5. IF a postcode provided in Disclosure UK proved to be related to the trust after websearch on the location and institution name, ’different postcodes but same organisation’ AND ’no action’ are written in the row in the ’Different organisations?’ and ’Any action in relation to differences between original and standardised postcodes?’ columns respectively.
   6. IF a postcode provided in Disclosure UK is proved to be related to a different trust than that identified from the database, after websearch on the location and institution name, the new standardised trust name is inserted into the “**Standardised NHS trust name - 3rd round column”**
      1. There were many cases in which the given address belonged to a different trust than what was provided in Disclosure UK. In those cases, the correct trust name was inputted into this column.
      2. The standardised postcode also needed to be changed to the one that relates to the new (correct) trust
      3. AND ’different postcodes but same organisation’ and ’no action’ were added to the row
   7. IF a postcode provided in Disclosure UK is proved to be related to other organisations (e.g. a clinical commissioning group, private company), was is mentioned in the comments column, weblink is added, and ’different organisation’ and ’remove’ is written in the row
   8. IF a postcode provided in Disclosure UK cannot be found, or the relation between the trust and the postcode cannot be established reliably, ’need for additional websearch’ and ’unclear’ or ’remove’ is written in the row
5. **Shared business services**
   1. during the process of checking postcodes that were different from standardised postcodes, some postcodes were found to be related to third party (intermediary) organisations
   2. these postcodes were: WF3 1WE, BB3 0FG, CM8 2TL
   3. when these were found, the standardised trust name is pasted from the 1st column to the 4th **Shared Services** column
   4. ’different postcodes but same organisation’ and ’no action’ were also added to the row

Overall, the key principles of the data extraction process were to

- Include as many results as possible to avoid excluding any payments made to NHS trusts. Given the complexity of the information at hand, this level of accuracy could only be achieved via detailed qualitative searches, and not an automated search.
- Ensure that the data was correct and that the healthcare organisations were correctly identified
- Organise the dataset in a way that is clearly comprehensible by anybody and shows all the data cleaning steps that we have taken.
- Allow for the repetition of the steps we took by other researchers.

The data extraction and cleaning were undertaken separately for the yearly editions of the Disclosure UK. The datasets were subsequently integrated. This did not raise any issues as the datasets had the same structure.

The data extraction and cleaning was carried out between July 2019 and August 2020.

The protocol for data extraction was developed jointly by tow researchers, PO and ES. The data extraction and cleaning was carried out by one researcher, ES. Given the highly structured nature of the process it was deemed not necessary for other researchers to be involved other than to clarify any emerging issues and ensure consistency.

#### Further data processing – VAT adjustment

### 2. Drug company methodological notes

We extracted data on the VAT approach of the 163 drug companies disclosing their payments in Disclosure UK at any point between 2015 and 2018. The methodological notes for each company and each year were downloaded from the website of the Association of the British Pharmaceutical Industry (5). These reports are in the Portable Document (PDF) format explaining the methodological approaches of each drug company in submitting information to Disclosure UK. The reports usually included information on whether payments (or “the transfers of value) reported in the database includes the VAT or not. This section of the report was found by searching for the word “VAT”.

##### VAT adjustment in Disclosure UK database

In a separate Excel sheet we extracted information of the approaches to reporting VAT by categorising each drug company report for each year as follows:

- 1 - all payments inclusive of VAT (gross)
- 2- all payments exclusive of VAT (net)
- 3 - no single rule regarding VAT
- 4 - not applicable (e.g. no payments)
- 6 might be classified as exclusive of VAT (net) but no clear statement
- 5 can be interpreted as inclusive of VAT (gross) based on the overall statement on reporting of tax
- 99 – VAT approach unclear

We then added a ’VAT approach’ column to the aggregated **Drug company payments to NHS trusts in England (2015-18)** dataset and added the number category to all payments of each company.

1. Adjustment of payments based on VAT approach

For payments with VAT categories 1, 3, 5, and 99, we made the assumption that the payment value included VAT; therefore we deducted the tax by multiplying the value with 0.8. For payments with VAT categories 2 and 6, we made the assumption that the payment was exclusive of the VAT, therefore we did not adjust the payment.

The approach to data extraction and categorisation was consistent with previous research on Disclosure UK (1, 6).

##### Further data processing – Inflation adjustment

We obtained the consumer price index (CPI) from the Office of National Statistics (7) for each year starting from 2015 with a base value of 100. The payment values were expressed in the 2018 sterling.

### 3. NHS trust annual accounts

The annual accounts of each NHS trust have been downloaded from the trusts’ official websites for each year. Data on i) whether or not the trust is a foundational trust; and ii) the service profile of the trust was collected from these accounts. Trust were put into 5 categories of service proviles:

- 1: Acute trust (provides secondary health services);
- 2: mental health trust (provides health services for patients with mental health disorders);
- 3: ambulance trust (provides ambulance services);
- 4: community health trust (provides community health services);
- 5: integrated care trust (integrates multiple types of services)

The service profile of each trust was determined through a qualitative process, searching for the key words in the accounts, such as “community”, “acute”, “mental”, “ambulance” and “integrated” and looking for evidence of the type of service that the trust provides. The results were collected in an Excel sheet and merged with the Drug company payments to NHS trusts in England (2015-18) dataset.

## S2 Table List of NHS trust mergers, 2015-2018

| Old trust name | New trust name | Year of name change |
| --- | --- | --- |
| Frimley Park Hospital NHS Foundation Trust | Frimley Health NHS Foundation Trust | 2014 |
| Mid Staffordshire FT | University Hospitals of North Midlands | 2014 |
| Tameside Hospital NHS Foundation Trust | Tameside and Glossop Integrated Care NHS Foundation Trust | 2016 |
| North Essex Partnership University NHS Foundation Trust | Essex Partnership University NHS Foundation Trust | 2017 |
| South Essex Partnership University NHS Foundation Trust | Essex Partnership University NHS Foundation Trust | 2017 |
| Central Manchester University Hospitals FT | Manchester University NHS Foundation Trust | 2017 |
| University Hospital of South Manchester | Manchester University NHS Foundation Trust | 2017 |
| University Hospital of South Manchester NHS Foundation Trust | Manchester University NHS Foundation Trust | 2017 |
| Hinchingbrooke HealthCare NHS Trust | North West Anglia NHS Foundation Trust | 2017 |
| Peterborough & Stamford Hosp NHS Trust | North West Anglia NHS Foundation Trust | 2017 |
| Heart of England FT | University Hospitals Birmingham NHS Foundation Trust | 2017 |
| Colchester Hospital University NHS FT | East Suffolk and North Essex NHS Foundation Trust | 2018 |
| The Ipswich Hospital NHS Trust | East Suffolk and North Essex NHS Foundation Trust | 2018 |
| Liverpool Community Health NHS Trust | Mersey Care NHS Foundation Trust | 2018 |
| South Staffordshire and Shropshire Healthcare NHS FT | Midlands Partnership NHS Foundation Trust | 2018 |
| Staffordshire and Stoke-on-Trent Partnership NHS Trust | Midlands Partnership NHS Foundation Trust | 2018 |
| Derby Teaching Hospitals NHS FT | University Hospital of Derby and Burton NHS Foundation Trust | 2018 |
| Hull and East Yorkshire Hospitals NHS Trust | Hull University Teaching Hospitals NHS Trust | 2019 |
| City Hospitals Sunderland NHS Foundation Trust | South Tyneside and Sunderland NHS Foundation Trust | 2019 |
| South Tyneside NHS Foundation Trust | South Tyneside and Sunderland NHS Foundation Trust | 2019 |
| Royal Liverpool and Broadgreen University Hospitals NHS Trust | Liverpool University Hospitals NHS Foundation Trust | 2019 |
| North Cumbria University Hospitals NHS Trust | North Cumbria Integrated Care NHS Foundation Trust | 2019 |
| Royal Liverpool and Broadgreen University Hospitals NHS Trust | Liverpool University Hospitals NHS Foundation Trust | 2019 |
| Aintree University Hospital NHS Foundation Trust | Liverpool University Hospitals NHS Foundation Trust | 2019 |
| Southend University Hospital NHS Foundation Trust | Mid and South Essex NHS Foundation | 2020 |
| University Hospitals Bristol NHS Foundation Trust | University Hospitals Bristol and Weston NHS Foundation Trust | 2020 |
| Weston Area Health NHS Trust | University Hospitals Bristol and Weston NHS Foundation Trust | 2020 |
| Black Country Partnership NHS Foundation Trust | Black Country Healthcare NHS Foundation Trust | 2020 |

## S3 Table Drug companies committing to payment disclosure and making payments to NHS trusts, 2015-2018

|  | 2015 | 2016 | 2017 | 2018 | All years |
| --- | --- | --- | --- | --- | --- |
| Number of drug companies committing to disclose payments | 109 | 113 | 115 | 122 | 163 |
| Number of drug companies making payments to NHS trusts | 83 (76.15 %) | 82 (72.57%) | 91 (79.13%) | 96 (78.69%) | 116 (71.17%) |

We understood drug companies committing to disclose payments as companies publishing their “methodological notes” on the Disclosure UK website.

## S4 Table Drug company non-R&D payments to HCOs in England, 2015-2018

|  |  | 2015 | 2016 | 2017 | 2018 | Total |
| --- | --- | --- | --- | --- | --- | --- |
| HCOs in England | **Number of payments** | 16,866 | 19,690 | 20,392 | 20,003 | 76,951 |
|  | **Value of payments** | £44,430,377.21 | £54,103,473.64 | £63,729,330.03 | £58,812,179.87 | £221,075,360.80 |
| NHS trusts in England | **Number of payments** | 6,517 (38.64) | 8,200 (41.65) | 8,239 (40.40) | 7,897 (39.48) | 30,853 (40.09) |
|  | **Value of payments** | £14,668,424.16 (33.01) | £16,245,811.25 (30.03) | £15,452,017.40 (24.25) | £13,887,169.05 (23.61) | £60,253,421.86 (27.25) |

## S5 Table NHS trusts receiving different categories of payments, 2015-2018

| Payment category | Number of NHS trusts (% of the total number of trusts receiving payments) | | | | | |
| --- | --- | --- | --- | --- | --- | --- |
|  | **2015** | **2016** | **2017** | **2018** | **At least once between 2015 and 2018** | **In all years between 2015 and 2018** |
| Grants and donations | 184 (85.98) | 194 (88.18) | 186 (86.92) | 171 (81.43) | 219 (93.59) | 146 (62.39) |
| Contributions to costs of events | 208 (97.2) | 209 (95) | 196 (91.59) | 201 (95.71) | 226 (96.58) | 181 (77.35) |
| Fees for service and consultancy | 82 (38.32) | 90 (40.91) | 114 (53.27) | 129 (61.43) | 169 (72.22) | 43 (18.38) |
| Joint working | 41 (19.16) | 31 (14.09) | 47 (21.96) | 36 (17.14) | 81 (34.62) | 8 (3.42) |

**Notes**

The denominators for individual years were the total number of NHS trusts receiving payments each year.

The denominator for all years was the total number of NHs trusts receiving payments.

## S6 Table Ordinary Least Squares and Random Effects explanatory models on payment numbers per NHS trusts per year

|  | REM (1) | REM (2) | REM (3) | REM (4) |
| --- | --- | --- | --- | --- |
| Mental health trusts | **-2.325*** (-0.289)** | **-2.321*** (-0.288)** | **-2.385*** (-0.282)** | **-2.385*** (-0.283)** |
| Ambulance trusts | **-3.342*** (-0.453)** | **-3.361*** (-0.452)** | **-3.400*** (-0.443)** | **-3.400*** (-0.444)** |
| Community health trusts | **-1.902*** (-0.243)** | **-1.910*** (-0.243)** | **-1.903*** (-0.236)** | **-1.903*** (-0.237)** |
| Integrated health trusts | **-0.754*** (-0.177)** | **-0.742*** (-0.177)** | **-0.742*** (-0.175)** | **-0.742*** (-0.175)** |
| Foundation_trusts |  | -0.207 (-0.162) | -0.211 (-0.161) | -0.211 (-0.161) |
| East England |  |  | -1.059* (-0.593) | -1.059* (-0.594) |
| East Midlands |  |  | 0.051 (-0.301) | 0.051 (-0.301) |
| East of England |  |  | 0.509* (-0.306) | 0.509* (-0.306) |
| North East |  |  | 0.097 (-0.296) | 0.097 (-0.297) |
| North West |  |  | 0.018 (-0.253) | 0.018 (-0.253) |
| South East |  |  | **0.740*** (-0.282)** | **0.740*** (-0.282)** |
| South West |  |  | 0.532* (-0.29) | 0.532* (-0.29) |
| West Midlands |  |  | 0.107 (-0.274) | 0.107 (-0.274) |
| 2016 |  |  |  | **0.154*** (-0.045)** |
| 2017 |  |  |  | 0.085* (-0.045) |
| 2018 |  |  |  | -0.019 (-0.045) |
| Constant | **3.391*** (-0.112)** | **3.529*** (-0.155)** | **3.336*** (-0.214)** | **3.281*** (-0.216)** |
|  |  |  |  |  |
| Observations | 936 | 936 | 936 | 936 |
| R2 | 0.133 | 0.135 | 0.159 | 0.173 |
| Adjusted R2 | 0.129 | 0.13 | 0.147 | 0.158 |
| F Statistic | 142.845*** | 145.328*** | 173.667*** | 191.987*** |

Note: Significance levels are *p<0.1; **p<0.05; ***p<0.01. P values below 0.05 are typed in bold. Reference categories are acute trusts, non-foundation trusts, London and 2015.

# Appendices

1. Ozieranski P, Csanadi M, Rickard E, Tchilingirian J, Mulinari S. Analysis of Pharmaceutical Industry Payments to UK Health Care Organizations in 2015. JAMA Network Open. 2019;2(6):e196253-e.

2. NHS England. Authorities and Trusts 2020 [Available from: <https://www.nhs.uk/servicedirectories/pages/nhstrustlisting.aspx>.

3. NHS England. Authorities and Trusts 2022 [Available from: <https://www.nhs.uk/servicedirectories/pages/nhstrustlisting.aspx>.

4. NHS England. Hospital List 2020 [Available from: <https://www.nhs.uk/Services/Pages/HospitalList.aspx?chorg=Hospital>.

5. ABPI. Disclosure UK 2020 [Available from: <https://www.abpi.org.uk/our-ethics/disclosure-uk/>.

6. Saghy E, Mulinari S, Ozieranski P. Drug company payments to General Practices in England: Cross-sectional and social network analysis. PLOS ONE. 2021;16(12):e0261077.

7. Office for National Statistics. CPIH INDEX 2022 [Available from: <https://www.ons.gov.uk/economy/inflationandpriceindices/timeseries/l522/mm23>.
