# Supplementary material for: Pharmaceutical industry payments to NHS trusts in England: A four-year analysis of the Disclosure UK database
Source: PLoS One. 2023 Nov 1;18(11):e0290022. doi: 10.1371/journal.pone.0290022 (PMC10619808; doi:10.1371/journal.pone.0290022)
Supplement: S1 Data — (DOCX) [file pone.0290022.s002.docx]

# Online supplements

## Online supplement 1 – Excel spreadsheet with drug company payments to NHS trusts in England, 2015-2018 (https://doi.org/10.6084/m9.figshare.21316944)

## Online supplement 2 – Excel spreadsheet with drug company payments to HCOs in England other than NHS trusts, 2015-2018 (https://doi.org/10.6084/m9.figshare.21316944)

## Online supplement 3 – R codes used to conduct calculations ([https://github.com/esaghy/NHS_Trust_analysis](https://eur01.safelinks.protection.outlook.com/?url=https%3A%2F%2Fgithub.com%2Fesaghy%2FNHS_Trust_analysis&data=05%7C01%7Cpo239%40bath.ac.uk%7C851bed3844e64daf377b08daac5e0adb%7C377e3d224ea1422db0ad8fcc89406b9e%7C0%7C0%7C638011817147605332%7CUnknown%7CTWFpbGZsb3d8eyJWIjoiMC4wLjAwMDAiLCJQIjoiV2luMzIiLCJBTiI6Ik1haWwiLCJXVCI6Mn0%3D%7C3000%7C%7C%7C&sdata=bf6h3wre3goGiUbttubVXBp3VQPPj72hVwfKt86JYNM%3D&reserved=0))
